# The Approximate Subcutaneous LD_50_ and Associated Lesions Induced by Ivalin, Extracted and Purified from *Geigeria aspera* Harv., in Sprague–Dawley Rats

**DOI:** 10.3390/molecules31030478

**Published:** 2026-01-29

**Authors:** Sara Locke, Christo Botha, Sarah Clift, Antoinette Lensink

**Affiliations:** 1 Department of Paraclinical Sciences, Faculty of Veterinary Science, University of Pretoria, Pretoria 0110, Gauteng, South Africa; 2 Department of Anatomy and Physiology, Faculty of Veterinary Science, University of Pretoria, Pretoria 0110, Gauteng, South Africa; sarah.clift@up.ac.za (S.C.); antoinette.lensink@up.ac.za (A.L.)

**Keywords:** *Geigeria aspera*, Vermeersiekte, sesquiterpene lactones, ivalin, rodent, subcutaneous median lethality, histopathology, ultrastructure, toxicodynamics

## Abstract

“Vomiting disease” in ruminants is one of the most economically significant phytotoxicities in South Africa and is caused by chronic ingestion of sesquiterpene lactone compounds present in plants of the *Geigeria* genus. Affected livestock demonstrate mortality due to actin and myosin damage in the striated musculature; however, a validated parental-exposure laboratory animal model would be useful for further study of the toxicodynamics. We exposed Sprague–Dawley rats to ivalin in a sequential dosing procedure and evaluated clinical signs, mortality, histopathology and muscle ultrastructure. Three of the five exposed rats died acutely, and a maximum likelihood estimate method was used to calculate a Median Lethality (LD_50_) of 135.4 mg/kg Body Weight (BW). Striated muscle in exposed rats showed only minimal and inconsistent histopathological and ultrastructural changes. Subcutaneous ivalin exposure causes acute mortality with minimal muscle pathology, contrasting with the more protracted muscular disease seen in ruminants after plant ingestion. This suggests toxicity by parenteral exposure is due to another mechanism, most likely mitochondrial energy pathway disturbances. Whilst subcutaneously exposed rats do not appear to provide a suitable model for oral sesquiterpene lactone exposure in ruminants, this study provides a starting dose for further investigation of plant extracts in both species.

## 1. Introduction

Plant toxicoses in livestock contribute significantly to agricultural economic losses in South Africa. Vomiting disease or “vermeersiekte” in sheep and cattle, caused mainly by ingestion of *Geigeria* plant species containing various sesquiterpene lactones, is one of the most important of these. Vermeersiekte causes striated muscle pathology, with resultant oesophageal dilatation, regurgitation of ruminal content, as well as stiffness and paralysis. The ensuing production losses and mortality cause severe financial losses to farmers [1,2].

The histopathology and ultrastructural lesions of vermeersiekte are well described and include myofibre vacuolisation and necrosis due to disruption of myosin and actin filaments in the heart, diaphragm, oesophagus and skeletal muscle. Mitochondrial swelling and cristae loss also occur [3,4]. However, despite cytological understanding of muscle pathology, the precise toxicodynamic mechanism of action is not known.

**Figure 1:**
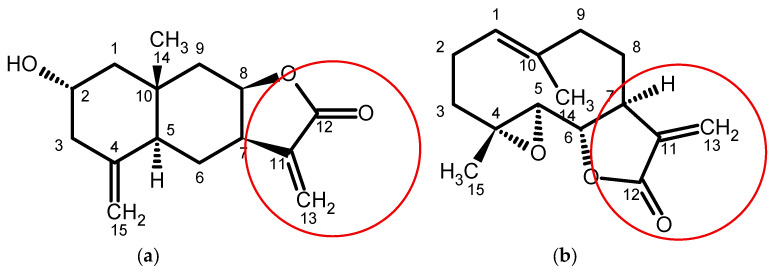
Chemical structure of the sesquiterpene lactones ivalin (**a**) and parthenolide (**b**). Note the presence of the exocyclic α-methylene-γ-lactone double bond at C-11 in both molecules (circled) [5,9].

Cytotoxicity of C-15 sesquiterpene lactones such as isogeigerin acetate, ivalin and geigerin, isolated from *Geigeria* spp., has been demonstrated in vitro in murine cell lines [5,6]. The toxicity of these compounds is ascribed to various functional groups, but most consistently the double bond exocyclic-methylene group conjugated to the γ-lactone of the carbocyclic skeleton [7,8]. Therefore, sesquiterpene lactone compounds possessing one or more of these functional groups, such as ivalin and parthenolide (Figure 1), are notably more toxic than those without, such as geigerin [5,6]. Recently, in vitro studies suggest that disruption of desmin intermediate cytoskeletal filaments may underlie muscle pathology in vermeersiekte, but this has not been validated in vivo [5,9].

As such, besides the avoidance of extended grazing on *Geigeria*-affected fields, no effective treatments are available. Efforts to understand the toxicodynamics of vermeersiekte in ruminants are further hampered by variability in oral toxicity and the need for large volumes of plant material required to dose sheep [1].

A validated rodent model following parenteral exposure would therefore be practically and economically valuable for further study of sesquiterpene toxicity and toxicodynamics, and to validate in vitro findings. The toxicity of sesquiterpene lactones from *Geigeria* spp. has also not been determined in rodents. The aim of this study was to determine the LD_50_ of ivalin, extracted and purified from *Geigeria aspera* Harv., in Sprague–Dawley rats, and to assess the correlation of muscle toxicosis between ruminants and rats when exposed by different routes.

## 2. Results

### 2.1. Clinical Signs and Mortality

Table 1 outlines individual ivalin doses for each rat and overall clinical observations. All exposed rats developed significant subcutaneous swelling from around 30 min after exposure, and in the animals that survived longer than 24 h, dermal irritation and self-traumatisation at the injection site.

In rat 1 (ivalin dose of 123 mg/kg BW), initial signs suggested recovery; however, after 48 h, the animal self-traumatised severely over the abdomen and lost more than 10% body weight. The animal was euthanised on humane grounds on day 4 following exposure. In accordance with the Organisation for Economic Co-operation and Development (OECD) Test Guideline (TG 425), the observation period following exposure was therefore, increased from 48 to 96 h.

Rat 2 (ivalin dose of 164 mg/kg BW) and rat 6 (ivalin dose of 219 mg/kg BW) became profoundly depressed and anorexic and died within 24 h following exposure.

Rat 3 (ivalin dose 123 mg/kg BW) and rat 4 (164 mg/kg BW) were both depressed following exposure but recovered and were clinically normal at euthanasia at the end of the study.

Mortality in rat 6 triggered a stopping criterion in OECD TG 425, and the study was concluded.

In control animals, there was no swelling at the exposure site. None of these animals exhibited signs of toxicity or dermal irritation. All displayed stable weight trends and were euthanised, as scheduled, 14 days after dosing.

### 2.2. Median Lethal Dose (LD_50_)

The LD_50_ output of the AOT425 statpgm software for subcutaneous ivalin exposure in Sprague–Dawley rats was estimated to be 135.4 mg/kg BW.

### 2.3. Macroscopic Pathology

External lesions in the rats were limited to moderate subcutaneous oedema associated with the self-inflicted traumatic skin lesions noted clinically.

### 2.4. Histopathology

In both exposed and control animals, the triceps brachii and gluteus maximus striated muscles exhibited mild to moderate congestion, often accompanied by small focal to multifocal haemorrhages. These changes were interpreted as agonal.

#### 2.4.1. Striated Muscle

**Figure 2:**
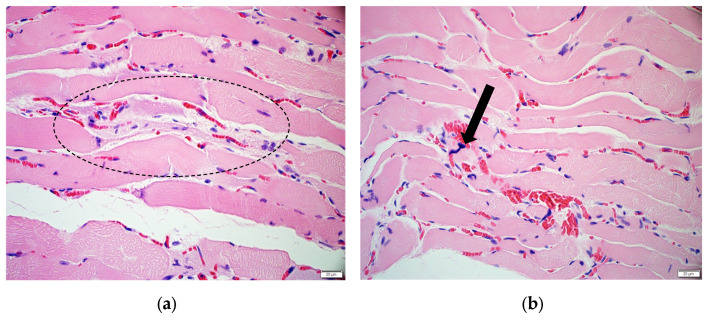
Myofibre findings in the diaphragm of ivalin-exposed rat 1. (**a**) Segmental myofibre vacuolisation and lysis associated with mild macrophagic infiltration (dashed oval) (H&E ×40). (**b**) Enlarged, irregular, hyperchromatic nuclei (arrow) (H&E ×40).

In rat 1 (ivalin dose 123 mg/kg BW) and to a lesser degree in rat 6 (ivalin dose 219 mg/kg BW), the diaphragm exhibited mild multifocal vacuolisation within the myofibre sarcoplasm, typically associated with segmental myofibre enlargement and loss of cross-striations. Multifocal lytic changes were detected in a few myofibre segments, accompanied by sarcoplasmic fragmentation, and sparse infiltration of macrophages (Figure 2a). Additionally, some myonuclei appeared irregularly enlarged and darkly stained, with unstructured chromatin; the nuclear membranes were disrupted, and the nuclear content appeared lysed. Increased numbers of internal myonuclei were observed (Figure 2b).

**Figure 3:**
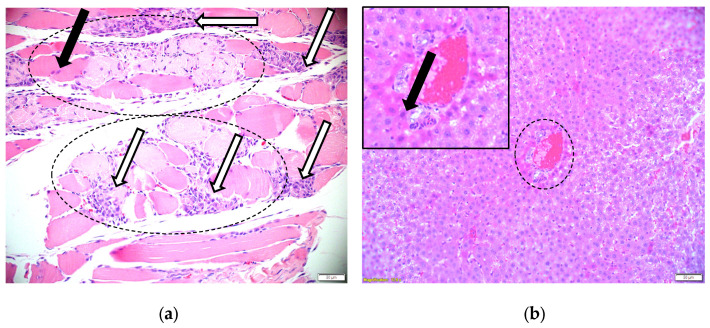
Skeletal muscle and hepatic changes in ivalin-exposed rat 2. (**a**) Transverse section of *Musculus triceps brachii* demonstrating mild to moderate myofibre swelling (dashed ovals), sarcoplasmic hypereosinophilia (black arrow), myofibre fragmentation/lysis with prominent macrophagic infiltration (white arrows), and sarcolemmal nuclear proliferation. (H&E ×20). (**b**) Periportal hypereosinophilia (dashed oval) and pyknotic nuclei (arrow in insert) (H&E ×20).

In rat 2 (ivalin dose 164 mg/kg BW), the myocardium just beneath the subendocardium contained rare, small foci of myofibre lysis with macrophage infiltration and minimal early fibrous repair. Skeletal muscle from the same rat exhibited moderate multifocal segmental necrosis with clear myofibre fragmentation, accompanied by macrophage infiltration and satellite cell activation (Figure 3a).

No changes were noted in the striated muscle of control animals.

#### 2.4.2. Liver

In rats 2 (ivalin dose 164 mg/kg BW) and 6 (ivalin dose 219 mg/kg BW), scattered periportal hepatocytes, particularly in the limiting plate, appeared hypereosinophilic with misshapen, pyknotic nuclei, indicating early cellular injury (Figure 3b). Additionally, the liver of rat 2 displayed moderate congestion, and rat 6 had karyorrhectic nuclear debris scattered in the sinusoids and periportal interstitium. No changes were noted in the liver of control animals.

### 2.5. Transmission Electron Microscopy (TEM)

**Figure 4:**
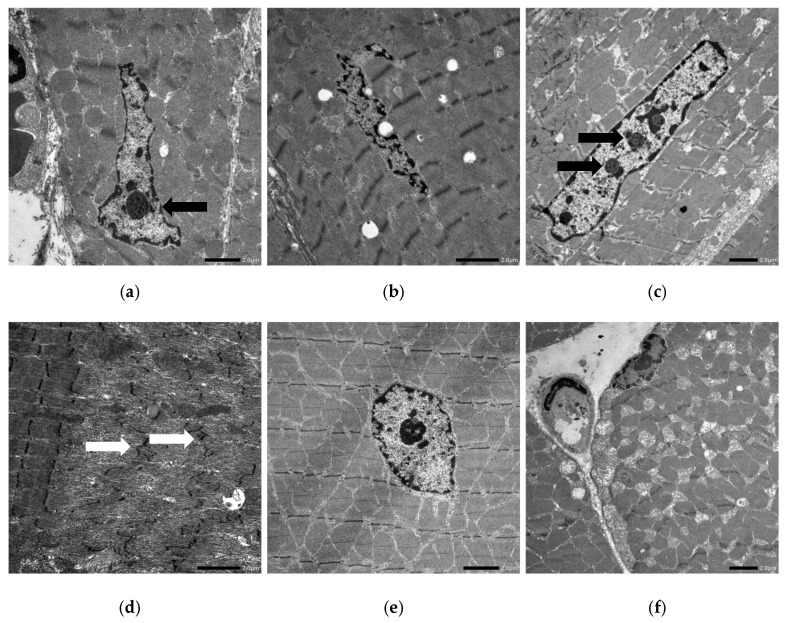
TEM of ivalin-exposed rats. (**a**–**c**) Cardiac muscle from rat 1 (**a**), rat 2 (**b**) and rat 6 (**c**) showing nuclear elongation (**a**–**c**), an irregular nuclear profile (**a**,**b**), chromatin marginalisation (**b**) and prominent nucleoli (**a**,**c**, black arrows). (**d**) Diaphragm of rat 4 showing z-line streaming (white arrows). (**e**) Skeletal muscle of a rat 4 showing centralisation of the nucleus. (**f**) Skeletal muscle of a rat 3 showing an increased number of mitochondria. One Bar = 2 µm.

In exposed rats, the heart myonuclei were elongated (particularly in rats 1 and 6), with an irregular profile (rats 1, 2 and 6), chromatin condensation and marginalisation, and multiple prominent nucleoli (rats 1, 2, 3 and 6) (Figure 4a–c). Small degrees of myofibrillar degeneration and dilation of the sarcoplasmic reticulum were observed.

In the diaphragm, some Z-line streaming is seen in rats 3 and 4 (Figure 4d). In the skeletal muscle of exposed rats, nuclear changes such as abnormal central positioning (rats 1, 4 and 6) (Figure 4e), elongation (rats 3 and 4) and condensation and margination of the chromatin (all rats) were observed. A marked increase in the number of mitochondria was also observed in skeletal muscle from some of the exposed rats (rats 1, 3, 4, and 6) (Figure 4f).

## 3. Discussion

The starting dose of ivalin for rats in this study (123 mg/kg BW) was based on a pilot study in mice [10]. The subcutaneous LD_50_ of ivalin (extracted and purified from *Geigeria aspera* Harv.) in rats was estimated by the maximum likelihood method in the OECD Up and Down Procedure (UDP) at 135.4 mg/kg. This methodology aims to minimise animal use, in accordance with the 3Rs principle (Replace, Reduce, Refine) for the use of animals in research. It allows compounds to be ranked and classified according to the Globally Harmonised System for the classification of chemicals which cause acute toxicity [11], and is therefore an approximate method. For a more robust value of LD_50_, with confidence intervals, methods such as the linear probit model could be used.

The median lethality reported by this study is similar to that of other sesquiterpene lactones in mice, though the exposure routes are different. Kim et al. (1980) reported intraperitoneal median lethal doses in female albino mice ranging from 3.08 to >200 mg/kg BW for different sesquiterpene compounds, isolated from *Helenium* and *Hymenoxys* spp. [12]. Mexicanin-E (LD_50_ 3.08 mg/kg BW) and helenalin (LD_50_ 9.86 mg/kg BW), both containing a cyclopentenone and a α-methylene-γ-lactone alkylating functional group, had toxicity levels of c. 10–60 times that of other sesquiterpene lactones containing only one alkylating centre, such as psiltropin (LD_50_ of 112.25 mg/kg BW), hymenoxon dimethyl ether (LD_50_ of 141.42 mg/kg BW) and tenulin (LD_50_ of 184.65 mg/kg BW). Ivalin, with one conjugated α-methylene-γ-lactone [6]; therefore, has an LD_50_ more consistent with psilotropin, hymenoxon dimethyl ether, and tenulin. Parthenolide, originally extracted from the feverfew plant (*Tanacetum parthenium)* and a sesquiterpene lactone of interest as an anti-cancer agent, demonstrated oral median lethality in Swiss albino mice of *c*. 200 mg/kg BW and is further discussed below [13].

Histopathology of the striated musculature revealed small, isolated foci of myofibre change in exposed rats compared to the controls. These changes were most evident in the diaphragm of rat 1, the myocardium of rat 2, and to a lesser extent, in the *Musculus triceps brachii* of rat 2, where only a single focus was identified. Observed changes included myofibre vacuolisation, enlargement, loss of cross-striations, and occasional mild lysis with macrophagic infiltration and early regeneration. Nuclear changes included centralisation, enlargement, subtle membrane irregularity, and loss of normal chromatin detail, with chromatin appearing clumped, smudged or diffusely hyperchromatic. These findings were confirmed by TEM in the cardiac tissue of rat 2, although only the nuclear changes were observed. However, muscle injury related to sesquiterpene lactone exposure might have been expected in rat 1, euthanised 4 days after dosing, but would not have been expected in rat 2, which died acutely overnight. The biological significance of the alterations seen in rat 2 therefore remains uncertain. Rats 3 and 4, both surviving to the study endpoint, showed no notable histopathology and only mild non-specific ultrastructural changes in the nuclei and mitochondria of the skeletal muscle, compared with controls.

Whilst TEM was not performed on hepatic tissue, histopathology in rats 2 and 6 indicated mild and early hepatocyte injury with nuclear pyknosis, hypereosinophilia and occasional karyorrhectic debris. Though this injury is consistent with findings in vermeersiekte-affected sheep [3,4], it was much less pronounced and not demonstrated in all exposed rats.

In domestic sheep and cattle affected by vermeersiekte, the disease is one of chronic striated muscle damage, with mortality typically resulting from aspiration pneumonia and progressive debilitation. Histopathology and ultrastructural changes in myofiber necrosis and inflammatory cell infiltration are notable and consistent, and fit with this clinical picture [3,4]. In this study, mortality occurred acutely, and lesions were either absent or mild, infrequent and inconsistent. Acute single-dose parenteral exposure of rats to purified sesquiterpene lactones therefore, it does not appear to provide a viable laboratory animal model for further study of more protracted *Geigeria* spp. ingestion in ruminants.

A possible reason for these different clinical and pathological outcomes may be related to differences in the exposure route. Although kinetic data on sesquiterpene lactones are limited, a recent review indicates that while these compounds are highly lipophilic and therefore potentially well absorbed orally, they are extensively metabolised by both phase 1 and 2 enzyme systems in the gastrointestinal tract, and potentially therefore, also the liver. Furthermore, gastrointestinal P-gp (P-glycoprotein) transporter efflux proteins contribute to variable or reduced oral absorption. Interspecies anatomical and physiological variation may also play a role [14]. Therefore, pre-systemic elimination (including possible ruminal microbial degradation), combined with chronic plant ingestion over several weeks, may lead to prolonged, but lower levels of exposure to sesquiterpene lactones in sheep, resulting in different toxicodynamics and the observed muscle pathology.

Kinetic differences between exposure routes for sesquiterpene lactones is also suggested to occur in the murine study by Kim et al. (1980), where lower median lethality for subcutaneous (1.59 mg/kg BW) compared to intraperitoneal exposure (3.08 mg/kg BW) for the compound mexicanin-E, suggests slightly greater bioavailability from subcutaneous compared to intra-peritoneal exposure, from which pre-systemic elimination also occurs [12]. Though a subcutaneous LD_50_ for parthenolide in either mice or rats is not available, the oral median lethality of this sesquiterpene lactone in mice is c. 200 mg/kg [12]. Parthenolide has both a conjugated α-methylene-y-lactone and an epoxide functional group [15]; however, the oral toxicity is much lower than that of intra-peritoneal mexicanin-E, containing two functional groups. This difference may also be due to presystemic elimination in the gastrointestinal tract.

In vitro studies in murine myoblastic cell lines suggest that desmin disruption may underlie actin and myosin disruption following sesquiterpene lactone exposure [5,9]. In myocytes, desmin forms a three-dimensional ‘net-like’ extra-sarcomeric scaffold that connects the contractile apparatus at the Z-discs with the subsarcolemmal cytoskeleton, as well as the nuclei and other cytoplasmic organelles, including the mitochondria [16,17]. Diseases that phosphorylate desmin precede and promote the breakdown of myofibrils [18,19]; however, to date, this toxicodynamic mechanism has not been demonstrated in vivo in either laboratory animals or ruminants.

Desmin plays a crucial role in mitochondrial homeostasis, and the coupling of mitochondria and the sarcomeres via desmin has been recognised as key for optimal energy use in myocytes, particularly in cardiac muscle [20]. Mitochondrial abnormalities are an early indicator of defective muscle, and include enlargement, decreased cristae density and vacuolisation [21]. Though the exact mechanism of interaction of desmin with mitochondrial structure and functioning is not yet fully elucidated, desmin interacts with voltage-gated anion channels, a component of cristae organisation called motilin and a subunit of complex V or ATP-synthase [21]. Furthermore, both desmin loss and post-transformational misfolding toxicity and associated amyloidosis are damaging to mitochondria [22].

Mitochondrial abnormalities, including swelling and cristae lysis, have been noted in sheep affected by vermeersiekte [3]. Therefore, though desminopathy as a mechanism has not yet been fully evaluated in vivo, it is possible that desmin disruption and/or aggregation, with associated effects on both myofibrils and mitochondria, could be implicated in vermeersiekte in ruminants.

In rats in this study, except for an increase in muscle mitochondrial numbers in some exposed rats, no major differences in mitochondrial pathology were observed compared to control rats. It is, therefore, more plausible that acute ivalin toxicity relates directly to effects on energy metabolism. This is supported by studies by van Aswegen et al. (1979, 1982), which demonstrate that sesquiterpene lactone interference with mitochondrial oxidative phosphorylation could account for the acute deaths seen in rodents, and occasionally in sheep naturally exposed to *Geigeria* spp. of plants [23,24]. Sesquiterpene lactones have also been reported to disrupt glycolytic and mitochondrial energy pathways, including in studies investigating their anti-neoplastic potential [25,26,27,28,29,30,31,32].

Whilst subcutaneous exposure to purified plant extracts is not the usual route of exposure to plant-derived sesquiterpene lactones in ruminants, the calculated median lethality from this study could provide a starting dose for purified plant extract studies in sheep, potentially reducing the need for large dosing volumes of oral administration of plant material and with the added benefit of bypassing pre-systemic elimination. Ivalin exposure via oral and parenteral routes in both ruminant and rodent species would potentially enhance our understanding of sesquiterpene lactone kinetics and the pathogenesis of the muscle pathology in vermeersiekte. Further study into the effects of *Geigeria* spp.extracts on desmin is warranted as pharmacological targets for intervention in human desminopathies become more important [20]. As the subcutaneous administration of these compounds appears extremely irritant, intravenous exposure at lower starting doses could potentially reduce inflammatory effects at the site of exposure.

## 4. Materials and Methods

### 4.1. Study Design

An in vivo study model, according to the global gold-standard OECD guidelines for toxicity testing in animals. The study design used is detailed in OECD Test Guideline (TG) 425 for Acute Oral Toxicity in rodents, otherwise known as the “Up-and-Down Procedure” or UDP [11]. Subcutaneous exposure was chosen to evaluate differences in exposure routes between rodents and ruminants. Animals were dosed with ivalin, one at a time, in a stepwise fashion, at a minimum of 48 h intervals. The first animal received a dose 1 dose-step below the best estimate of the LD_50_, determined from a previous unpublished study in CD-1 mice of 164 mg/kg BW [10]. In OECD TG 425, a combination of stopping criteria is used to keep the number of animals low; the study stops when the first stopping criterion is met. A control group (n = 5) was used to monitor the health and welfare of test rats to ensure that the ability of the study to provide reliable results was not compromised, and to provide control samples for analysis.

### 4.2. Animals

This study was approved by the University of Pretoria Animal Ethics Committee and the Faculty of Veterinary Science Research Ethics Committee under project number REC 038-23.

Six-week outbred Sprague–Dawley female rats (n = 15) were randomly assigned to a study group (Table 2) and allowed a 14-day observation and acclimatisation period. Animals were housed in Type II 1284L rodent cages (Tecniplast, Via Maggio, Italy), room temperature was maintained at 22–24 °C, relative humidity was maintained between 40 and 60%, and lighting was artificial with the photoperiod set at 12 h light/12 h dark. Rats were weighed individually on the second day of arrival and weekly until dosing. On the day of dosing, animals were weighed again and then every second day, or as needed.

### 4.3. Ivalin Preparation and Dosing

Ivalin was previously extracted and purified from *Geigeria aspera* Harv. by Fouche et al. (2021) [6]. The structure, identified as LA02-86-1-(2), was deduced using 1 and 2D NMR spectroscopy and mass spectrometry, while the absolute configuration was determined for the first time by X-ray crystal diffraction [6]. The structure of ivalin is depicted above (Figure 1). Ivalin was weighed, according to the individual animal weight prior to dosing, and dissolved into 0.5 mL PEG 400 vehicle in a sterile 2 mL Eppendorf tube. Complete dissolution was facilitated by Vortex agitation (Heathrow Scientific, Vernon Hills, IL, USA) and a heated water bath (37 °C). An additional 0.1 mL of sterile water was added to the prepared ivalin solutions to reduce viscosity for ease of parenteral administration. The solutions were aspirated into 1 mL syringes for dosing.

Rats were dosed subcutaneously between the scapulae one at a time, in a step-wise fashion, according to Table 3. Initially, the time between dosing was set at 48 h, as prescribed by the OECD guidelines [11]. However, rat 1 did not die acutely, but rather deteriorated chronically over 4 days before being euthanised; therefore, the inter-dosing interval was extended to 4 days. Rats were furthermore observed for 14 days following dosing, as prescribed by the OECD guidelines. Any animal showing undue suffering was euthanised.

### 4.4. Observations and Sampling

#### 4.4.1. Clinical

Animals were observed continuously for the first 30 min following dosing and once hourly during the first 4 h thereafter. Following this, animals were observed twice daily for up to 14 days post-dosing. Signs of pain, stress and/or toxicity were assessed using the Rat Grimace Scale [33], as well as facility-recognised parameters, including staring coat, staining of the medial canthus of the eye, muscle tremors, stiffness, lethargy, weakness, appetite and weight loss, respiratory distress and recumbency.

#### 4.4.2. Necropsy and Tissue Sampling

All animals were subjected to a full necropsy. This included a detailed macroscopic/gross examination of the external surface of the body, orifices and the thoracic and abdominal cavities and their contents. The liver, oesophagus, diaphragm, cardiac and skeletal muscles (*Musculus triceps brachii* and *M. gluteus maximus*) were collected and divided for histopathology and TEM. Muscles were pinned to prevent contraction. Samples for light microscopy were routinely processed, sectioned and stained with haematoxylin and eosin (H&E). Samples for TEM underwent chemical fixation (glutaraldehyde), dehydration (propylene oxide), embedding in epoxy resin and ultrathin sectioning (microtomy) with a microtome for Transmission Electron Microscopy for TEM.

### 4.5. Data Analysis

#### 4.5.1. Clinical Signs and Mortality

Clinical signs of toxicity were recorded descriptively, and time to mortality (or euthanasia) was recorded in hours post-dosing. Weight changes were tabulated, and percentage weight losses calculated. Animals that were euthanised were included as deaths occurring due to ivalin exposure.

#### 4.5.2. Median Lethal Dose (LD_50_) Calculations

The LD_50_ was calculated using the maximum likelihood method. According to Dixon et al. (1991), the likelihood function is written as:

L = L_1_L_2_…L_n_,

where L is the likelihood of the experimental outcome, given µ (the mean), σ (the standard deviation) and n (the total number of animals tested) [34].

L*_i_* = 1 − F(Z*_i_*) if the *i*th animal survived, or L*_i_* = F(Z*_i_*) if the *i*th animal died

where F = cumulative standard normal distribution, Z*_i_* = [log(d*_i_*) − µ]/σ; d*_i_* = dose given to the *i*th animal and σ = the standard deviation in log units of dose.

An estimate of the LD_50_ is given by the value of µ that maximises the likelihood L [28]. An estimate of 0.125 was used for σ, corresponding to a slope of 8, following an unpublished pilot study in CD-1 mice which indicated a steep dose–response curve [10].

Maximum likelihood calculations and confidence interval calculations were performed using the Acute Oral Toxicity (OECD TG 425) Statistical Programme (AOT 425 StatPgm) Version: 1.0, 2001 https://www.oecd.org/env/ehs/testing/section4software.htm (accessed on 26 January 2026), downloaded from the U.S. Environmental Protection Agency website [35].

#### 4.5.3. Pathology, Desmin Immunohistochemistry and TEM

Macro- and histopathology, ultrastructural findings and desmin reactivity were qualitatively evaluated and described.

## Figures and Tables

**Table 1:** Individual dose and clinical outcomes in Sprague–Dawley rats exposed subcutaneously to ivalin.

Rat	Group	Weight at Dosing (g)	Dose of Ivalin (mg/kg BW)	Outcome	Time to Mortality (h)
1	Exposed	235	123	Died	96
2	Exposed	229	164	Died	<24
3	Exposed	213	123	Survived	-
4	Exposed	225	164	Survived	-
6	Exposed	239	219	Died	<24
11	Control	210	*	Survived	-
12	Control	231	*	Survived	-
13	Control	234	*	Survived	-
14	Control	225	*	Survived	-
15	Control	235	*	Survived	-

* Control animals received only the vehicle, Polyethylene Glycol 400 (PEG 400).

**Table 2:** Rat identification numbers per study group.

Study Group	Rat Numbers	Treatment
Exposed animals	1–10	Ivalin in PEG 400, according to OECD TG 425
Control animals	11–15	PEG 400

**Table 3:** Stepwise ivalin dosing in rats.

Dose Step Number from Starting Dose										
−5	−4	−3	−2	−1	Starting dose	+1	+2	+3	+4	+5
**Dose in mg/kg BW**										
29	39	52	69	92	123	164	219	290	390	520

## Data Availability

The original data presented in the study are openly available in FigShare at http://hdl.handle.net/2263/102980, accessed on 26 January 2026.

## Abbreviations

The following abbreviations are used in this manuscript:

LD_50_	Lethal Dose (50) or Median Lethal Dose
BW	Body Weight
OECD	Organisation for Economic Co-operation and Development
OECD TG	OECD Test Guideline
OECD UDP	OECD Up-Down-Procedure
PEG 400	Polyethylene Glycol 400
H&E	Haematoxylin and Eosin
TEM	Transmission Electron Microscopy
P-gp	P-glycoprotein
